# Finding social need-les in a haystack: ascertaining social needs of Medicare patients recorded in the notes of care managers

**DOI:** 10.1186/s12913-023-10446-2

**Published:** 2023-12-12

**Authors:** Paul R. Shafer, Amanda Davis, Jack A. Clark

**Affiliations:** Department of Health Law, Policy, and Management, School of Public Health, Boston University, 715 Albany Street, Boston, MA 02118 United States of America

**Keywords:** Patient care management, Case management, Social determinants of health, Medicare, Chronic Disease

## Abstract

**Background:**

Unmet social needs may impair health and access to health care, and intervening on these holds particular promise in high-risk patient populations, such as those with multiple chronic conditions. Our objective was to identify social needs in a patient population at significant risk—Medicare enrollees with multiple chronic illnesses enrolled in care management services—and measure their prevalence prior to any systematic screening.

**Methods:**

We partnered with Renova Health, an independent Medicare Chronic Care Management (CCM) provider with patients in 10 states during our study period (January 2017 through August 2020). Our data included over 3,000 Medicare CCM patients, representing nearly 20,000 encounters. We used a dictionary-based natural language processing approach to ascertain the prevalence of six domains of barriers to care (food insecurity, housing instability, utility hardship) and unmet social needs (health care affordability, need for supportive services, transportation) in notes taken during telephonic Medicare CCM patient encounters.

**Results:**

Barriers to care, specifically need for supportive services (2.4%) and health care affordability (0.8%), were the most prevalent domains identified. Transportation as a barrier to care came up relatively less frequently in CCM encounters (0.1%). Unmet social needs were identified at a comparatively lower rate, with potential housing instability (0.3%) flagged most followed by potential utility hardship (0.2%) and food insecurity (0.1%).

**Conclusions:**

There is substantial untapped opportunity to systematically screen for social determinants of health and unmet social needs in care management.

## Background

Social determinants of health (SDOH) have become prominent concerns for clinicians, health systems, and payers in their efforts to improve health, reduce health care costs, and achieve health equity [1]. Unmet social needs may impair health and access to health care, and thus are recognized as targets of intervention [2]. Intervening on unmet social needs holds particular promise in patient populations at high risk, such as those with multiple chronic conditions, for whom unstable housing, inconsistent nutrition, or inadequate transportation to clinics could be the difference between successful outpatient illness management and hospitalization [3]. However, intervention on social risks requires their efficient ascertainment in the course of providing services in addition to understanding their presentation and management to guide effective clinical strategies. As a result, attention has focused on “social informatics”, through the mining of social risk information contained in clinical records along with innovation in the design of electronic health records and strategies to improve the documentation of social risks [4, 5].

Clinical records are combinations of formally structured (e.g., defined fields for quantitative examination and lab values) and unstructured (e.g., verbal notes of findings and assessments) information, with the latter a likely source of social risk information. Natural language processing (NLP) offers tools for generating useful data for clinical practice and research from largely unstructured records, particularly data about patients’ social circumstances. NLP can be used to automate the identification and classification of social needs to ensure accurate and appropriate care coordination and referrals to community services [6–9]. NLP has been shown to be effective at identifying unmet social needs when combining clinical free-text notes and structured data, which effectively expedites identification and outreach for vulnerable patients, and may be a complement to qualitative interviews and systematic screening [6, 9, 10].

However, making good use of patient data for research and practice still presents methodological challenges relating to variation in their contents, the terms used, the meanings of those terms, and the ways in which records are kept for diverse clinical purposes that are typically at odds with the interests of researchers. Moreover, the inclusion of psychosocial information relevant to social needs by physicians has long been uneven, prompting calls for improved standard practices [11, 12]. At the same time, responsibilities for addressing and recording information about patients’ life circumstances in many settings have shifted to other members of patient care teams, especially care managers. The tasks associated with care management—e.g., ensuring patients’ timely engagement in care, coordinating referrals, monitoring adherence to prescribed regimens, and promoting patients’ self-management in their real-world settings—requires attention to those life circumstances and psychosocial aspects of illness and health behaviors. The notes produced by care managers in their practices may include relevant psychosocial and social risk information. Yet, they still present problems of translation and sharing with other members of the care team as well as researchers seeking to understand patients and their social needs.

In this study, we identify social needs in a patient population at significant risk—Medicare enrollees with multiple chronic illnesses enrolled in care management services. Our goals are to (1) assess the prevalence of unmet social needs, which we might expect to be high in this population but have not been widely documented, using natural language processing to ascertain their presence in care managers’ records, and (2) characterize the work of care management in addressing those needs.

## Methods

The specific setting for this study is an independent care management provider that is representative of a growing domain of clinical services. The Centers for Medicare and Medicaid Services (CMS) issued guidance in 2015, stating it would pay for chronic care management services provided to eligible Medicare enrollees—patients with two or more chronic conditions “that place the patient at significant risk of death, acute exacerbation/decompensation, or functional decline” [13]. The Chronic Care Management (CCM) model defined by CMS involves the establishment, implementation, revision, and monitoring of comprehensive care plans through monthly patient encounters of at least 20 min, with documentation in an electronic health record (EHR) to allow for clinical collaboration. These monthly encounters are focused on how patients are faring, providing psychosocial information that supplements what clinicians may observe in their encounters with patients [14]. However, they remain limited to what can be communicated over the telephone. Care managers may be employed directly by health care systems or provider groups, or alternatively (and increasingly), by independent providers of care management services that contract with health systems.

We partnered with Renova Health, an independent Medicare CCM provider with patients in 10 states (Alabama, Alaska, Georgia, Maryland, Minnesota, Missouri, New York, Ohio,  Tennessee, Virginia) during our study period. Our study data included all CCM patient encounter notes from January 2017 through August 2020 with anonymized patient identifiers, age, sex (male or female), ZIP code of residence, the conditions that qualified them for CCM, month and year of encounter, anonymized care manager identifier, and the free text notes associated with the encounter. All encounters were conducted over the telephone. These data from over 3,000 patients and nearly 20,000 encounters over a period of more than three years serve as the corpus of free text notes for our analysis, providing recurring interactions and deepening relationships between patients and care managers.

We used a dictionary-based natural language processing approach for our study, conducted in WordStat 9 from Provalis Research [15], which allowed us to make explicit coding and logic decisions. This approach also allowed us to test variants of our dictionary for each domain of interest, unlike a machine learning-based approach where coding and relative contribution of key words or phrases would be obscured and thematic discrimination may struggle due to low prevalence. Our dictionary (Table 1), in which each domain is defined by a set of key words and phrases, was developed based on reviewing existing literature for terms associated with unmet social needs and barriers to care, our own contributions, discussions with four Renova Health care managers (see Acknowledgments) about the terms that they use and when and how those terms are applied, and iterative changes after testing against manual coding with a 5% random subsample [16–18]. We selected six domains to ascertain the prevalence of during Renova Health care management encounters, three each representing unmet social needs and barriers to care—1) food insecurity, 2) housing instability, 3) utility hardship, 4) health care affordability, 5) need for supportive services, and 6) transportation. For key combinations of words (e.g., x AND y), we required them to appear in the same sentence to provide a stronger likelihood of them referencing the desired construct. For single key words or key words in combination through logic statements, the software automatically considers plural and related forms of the root word. We used presence of a key word or phrase (Boolean query) as our prevalence outcome, rather than a method such as TF-IDF (term frequency-inverse document frequency) [19, 20], given the relatively low prevalence of the domains of interest in these encounter notes.

**Table 1:** Key words and phrases by domain

Domain	Key words and phrases
*Unmet social needs*	
Food insecurity	(diet OR food OR nutrition) AND (expensive OR afford OR costly), food assistance, food bank, food banks, food insecurity, food pantry, food pantries, Meals on Wheels, MOW
Housing instability	(rent OR mortgage) AND (late OR default OR delinquent OR delinquency OR afford OR notice), landlord AND (late OR afford OR notice), default, evict, Faith in Action, foreclosure, homeless, housing, HUD, lodging, motel, Peoples Inc, shelter
Utility hardship	(power OR electric OR gas OR heat OR water OR AC) AND (expensive OR afford OR costly), air conditioner, air conditioning, disconnect, shut off
*Barriers to care*	
Health care affordability	(doctor OR hospital OR drug OR prescription OR medication OR rx OR test) AND (expensive OR afford OR costly), afford, costly, expensive, medical bill, medical bills
Need for supportive services	caregiver AND (difficult OR hard OR need OR expensive OR miss OR afford OR costly), able to leave, able to take, alone, have no one, need help, need assistance, safe
Transportation	(transportation OR transit) AND (concern OR assistance OR need OR help OR afford), (car OR truck OR vehicle) AND (repair OR assistance OR need OR help OR afford), (bus OR train OR subway OR shuttle) AND (need OR assistance OR help OR afford)

**Fig. 1:**
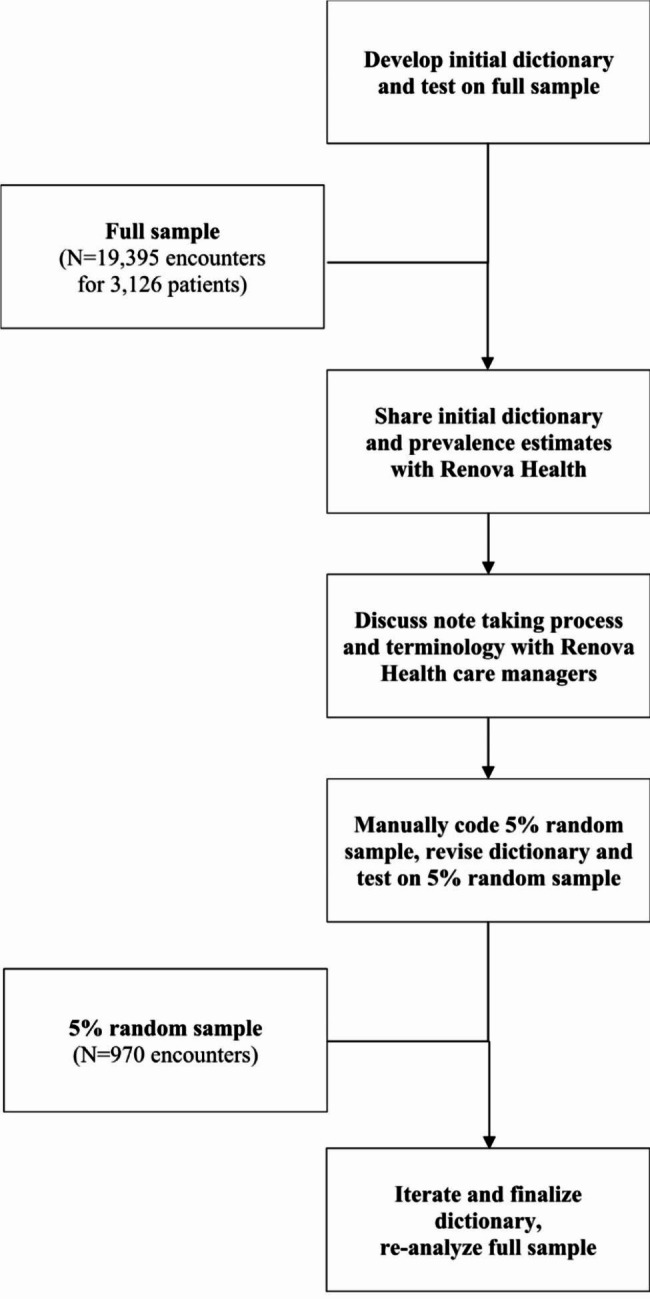
Flow diagram of dictionary development and analysis

Our dictionary-guided ascertainment is subject to error related to the relationships between search terms and actual references to social risks in the care managers’ notes. Hence, we evaluated the accuracy of our procedure by comparing its performance relative to manual coding of care managers’ notes and calculating test statistics—sensitivity, specificity, and positive predictive value. We first selected a 5% random sample of 970 encounter notes, which were coded by two of us (AD and JC), working independently. The coding identified the presence of any documented unmet social needs and barriers to care in each encounter note, without knowledge of the determinations produced by the algorithm. Their independent coding was compared using kappa statistics of agreement, resulting in very high agreement (kappa = 0.920 averaged across the six themes, ranging from 0.748 [need for supportive services] to 1.000 [utility hardship]). Identification in either coding was used to create a blended manual coding, which was then compared with the identification using the dictionary-based search algorithm to assess sensitivity, specificity, and positive predictive value. We iteratively refined our dictionary until the blended manual and algorithm yielded similar prevalence before applying to the full corpus of encounter notes. For the full corpus, we present prevalence of each domain in care manager encounter notes as identified using our dictionary-based search algorithm. Figure 1 provides a visual description of the steps in our dictionary development and analysis summarized above. Our study meets the Strengthening the Reporting of Observational Studies in Epidemiology (STROBE) guidelines for cross-sectional studies [21].

## Results

Our corpus of Medicare Chronic Care Management patient encounters from Renova Health consists of 3,126 patients with 19,395 telephonic encounters for the period of January 2017 to August 2020 (Table 2). The average age (at first encounter) of this patient population was 76.6 years and most were female (59.3%). To provide context on the health and qualifying conditions of the patient population included, we tabulated the prevalence of 17 potentially qualifying conditions for CCM, which should not be considered exhaustive [13]. At least two chronic conditions are required for participation in CCM. The most prevalent qualifying conditions for CCM (at first encounter), of those included, were hypertension (73.7%), hyperlipidemia (54.0%), diabetes (37.4%), hypothyroidism (15.8%), chronic obstructive pulmonary disease (11.8%), arthritis (10.9%), anxiety (10.9%), and depression (10.5%) with the remainder present in less than 10% of patients. Our data unfortunately did not contain other demographics relevant to social needs, such as race, ethnicity, education, or marital status.

**Table 2:** Patient characteristics, Renova Health, January 2017 to August 2020

Characteristic	
Age (at first encounter), mean	76.6
Sex, %	
Male	40.7%
Female	59.3%
Qualifying conditions for Chronic Care Management (at first encounter), %	
Alzheimer’s Disease and related dementias	0.6%
Anxiety	10.9%
Arthritis	10.9%
Asthma	3.6%
Atrial fibrillation	5.5%
Autism spectrum disorder	0.2%
Cancer	3.9%
Cardiovascular disease	6.4%
Chronic kidney disease	8.8%
Chronic obstructive pulmonary disease	11.8%
Depression	10.5%
Diabetes	37.4%
Heart failure	3.3%
Hyperlipidemia	54.0%
Hypertension	73.7%
Hypothyroidism	15.1%
Infectious diseases, such as HIV	0.0%
N (patients)	3,126
N (encounters)	19,395

Incidence of qualifying conditions is not mutually exclusive and therefore the percentages across the seventeen conditions included sum to over 100%

Our 5% random sample of encounter notes (Table 3) had very low prevalence of each domain as determined by the manual coding, with 19 or fewer positives, or 2% or less of encounters, for each domain (two for food insecurity, one for housing instability, zero for utility hardship, 10 for health care affordability, 19 for need for supportive services, and two for transportation). Thus, the information from test performance statistics was limited [22]. Very low underlying prevalence renders sensitivity uninformative, while zero prevalence (in the case of utility hardship) makes it incalculable. Conversely, specificity was very high—above 97.9% for all six domains—as might be expected. Yet, the prevalence estimates produced by the NLP in the 5% sample were very similar to those produced by manual coding.

**Table 3:** 5% random sample comparison of manual and NLP coding by domain, Renova Health, January 2017 to August 2020

Domain	Manual coding prevalence	Kappa statistic (for coder agreement)	NLP prevalence	Sensitivity	Specificity	PPV^1^	NLP prevalence (full corpus)
*Unmet social needs*							
Food insecurity	0.2%	0.973	0.2%	0.0%	99.8%	0.0%	0.1%
Housing instability	0.3%	0.960	0.2%	33.3%	99.9%	50.0%	0.3%
Utility hardship	0.0%	1.000	0.0%	–	100%	–	0.2%
*Barriers to care*							
Health care affordability	1.0%	0.867	0.8%	50.0%	99.7%	62.5%	0.8%
Need for supportive services	2.0%	0.748	2.6%	26.3%	97.9%	20.0%	2.4%
Transportation	0.2%	0.973	0.1%	50.0%	100%	100%	0.1%
*Any of the above*	3.4%	0.920	3.7%	–	–	–	3.9%

^1^ Positive predictive value

**Fig. 2:**
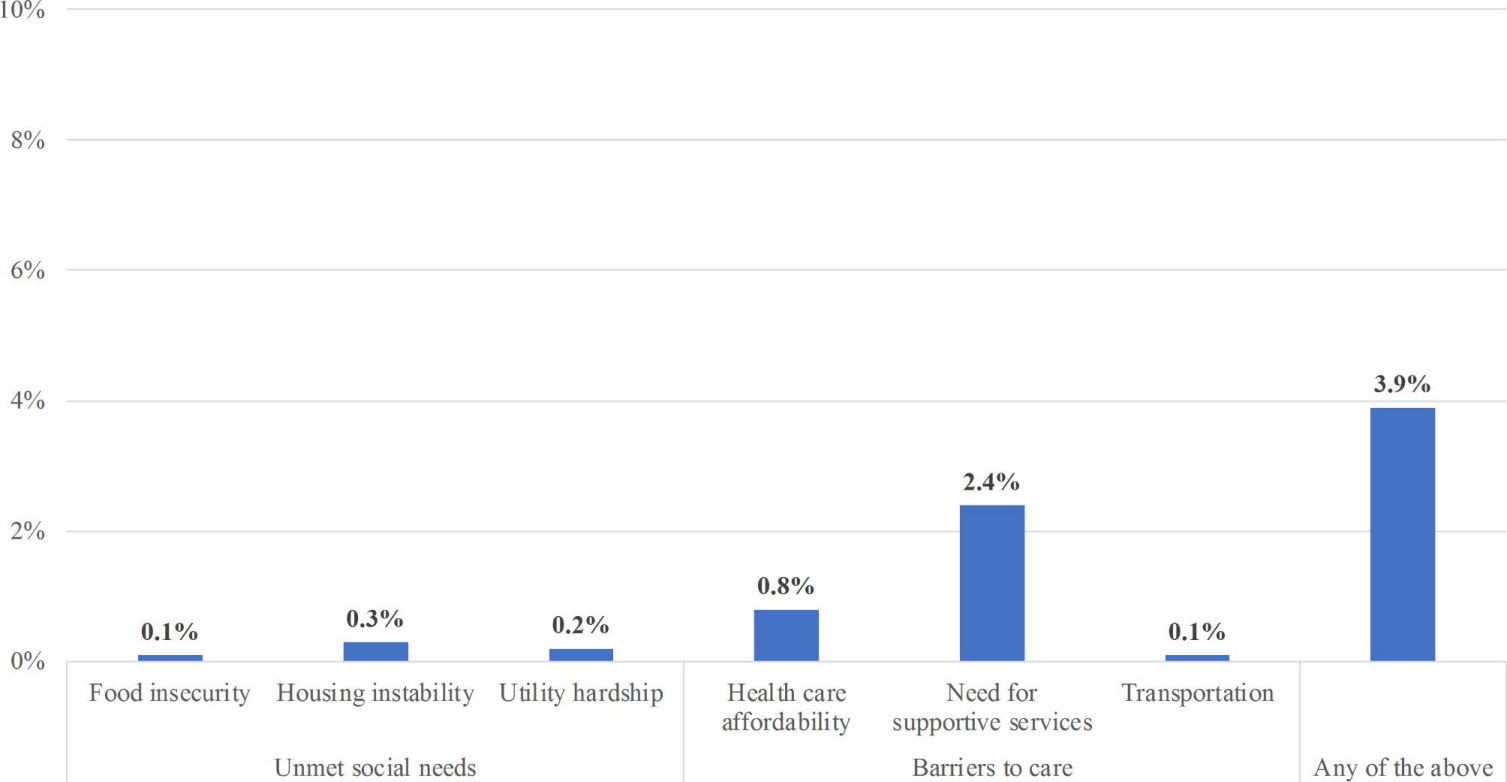
Prevalence of unmet social needs and barriers to care, Renova Health, January 2017 to August 2020

Barriers to care, specifically need for supportive services (2.4%) and health care affordability (0.8%), were the most prevalent domains identified in our corpus of CCM encounters (Table 3; Fig. 2). However, transportation as a barrier to care came up relatively less frequently in CCM encounters (0.1%) than the other two. Unmet social needs were identified at a comparatively lower rate in our corpus of CCM encounters, with potential housing instability (0.3%) flagged most followed by potential utility hardship (0.2%) and food insecurity (0.1%) (Table 3; Fig. 2).

## Discussion

Among over 3,000 Medicare CCM patients in 10 states, we found that care management encounters rarely resulted in documentation of unmet social needs—identification of barriers to care were relatively more common, particularly health care affordability and need for supportive services, but still rare. This imbalance may reflect the priorities of care managers as well as the needs of their clients, both patients and other providers, given the limited time available in a monthly telephonic encounter. They may focus on domains of care that they can do something about, such as connecting patients to supportive services or providing coupons or other assistance to defray medication costs, and/or patients may be unwilling or less willing to disclose challenges until trust is developed [23–25]. We demonstrate a practical strategy for mining care managers’ notes for barriers to care and unmet social needs, bringing care managers into the process to reflect on the terms that they use to document different findings. Ascertainment of social needs in care management records ought to be grounded in knowledge of how the producers of records practice, including how they record what they see, with awareness that different systems and providers will approach delivery of a model like CCM differently, creating considerable variation in the patient experience [26–28]. The difference in ascertained prevalence of barriers to care compared with unmet social needs may require a deeper level of inquiry and sensitive attention to what patients say. An important limitation of this, and all other work based on clinical notes, is that what we “observe” relies on secondhand records—i.e., written notes of a verbal encounter—that may not exhaustively represent the substance of the conversation. However, during a subsequent period when systematic screening for unmet social needs was introduced by Renova Health, they observed largely similar or lower prevalence, suggesting that care managers were suitably monitoring these risks and their notes could be considered a good source of information in the absence of systematic screening. Also, although our results may be generalizable and internally consistent within the Renova Health population of Medicare CCM patients, they may not generalize to the experience and social risks present within Medicare CCM patients as a whole. Another important consideration for this population is that despite their known needs (i.e., having at least 2 qualifying conditions), they also need to be willing and able to pay a $20 co-pay for care management services, which may select out those with lesser means that may have expressed greater unmet social needs. Qualitative research specific to Medicare CCM to understand care manager and patient perspectives on the program—how they approach these encounters, what is being prioritized (e.g., health and medical care versus social needs), and trust, for example—could help shed further light on and disentangle the intersecting challenges of selection bias (who can afford to and chooses to participate), what is documented (versus what is said), and what patients choose to share in this context.

The care managers said that while they may not inquire about social needs per se, they addressed and recorded relevant issues as they inquired about patients’ management of their prescribed medication regimens, attendance at scheduled clinic visits, and how they were generally taking care of themselves. For example, “Were they getting exercise and eating well?” Patients might disclose difficulties, explaining missing doses of their medications because of costs or note the high cost of groceries and budgets stretched to cover groceries, utilities, and medications in relation to the quality of their diet. Care managers said they responded to these disclosures by offering assistance: prescription discount coupons, advice on enrolling in patient assistance programs provided by pharmaceutical companies and help with the application forms, facilitating access to Meals on Wheels programs and food banks, and providing the name of a transportation service for clinic visits. One care manager described reviewing household budgets to find opportunities for relief, such as identifying utility costs that could be reduced with a program for elders in need, thus freeing money for groceries and medications. Their interests were practical; their job was to help with managing care. They identified issues they could help resolve with resources they knew were available. They would document these issues, but do so elliptically, such as “costs of medications” without mention “difficulty.” Terms such as “financial insecurity” were rarely used. Instead, the records included notes of what they did or the resources they provided. Along with “costs of medications” they might note “sent discount coupon” or “sent information about PAP (patient assistance program).” Diet difficulties would be recorded as “advised” or “encouraged [to contact] meals-on-wheels” or a specific local food bank by name.

There is considerable information about patients that cannot be captured in a short physician appointment. Care management can be another touch point for gathering information about patients’ social context; however, a key facilitator of this potential is deep integration of care management data with providers and/or health systems’ patient records [29]. Realizing this potential also requires processes that are not dependent on an individual care manager or clinician, creating opportunities for approaches like ours to be deployed to generate near real-time intelligence for use in dashboards and/or automatically starting referral mechanisms. It also reinforces that understanding how care managers and other non-clinicians convert verbal patient encounters into written documentation is key as we can only learn from and act on what is written down. Providing responsive and appropriate care for high-cost complex patients is necessary, but not sufficient, to improve health outcomes. Taking better advantage of ancillary providers holds promise, but requires overcoming considerable challenges to how information is shared and acted upon in a fragmented care landscape.

## Conclusions

Our findings demonstrate that notes from Medicare CCM encounters rarely uncover–or at least document–barriers to care or unmet social needs for enrolled patients, with the latter being comparatively far less prevalent. Given the high risk and high cost of this patient population, there would seem to be a substantial untapped opportunity to systematically screen for social determinants of health and unmet social needs in care management, making care managers into agents of change to improve population health [30–33]. However, next best approaches, like text analysis of clinical notes, could also powerful in generating social risk indicators. Variation in how payers and health systems use care management as well as how care managers engage with patients about their unmet social needs represent critical edges of research going forward, including close attention to the tools they use and how they work.

## Data Availability

The data underlying this article cannot be shared publicly as they are anonymized patient records shared by Renova Health under a data use agreement specific to this application.

## Abbreviations

CCM: Chronic Care Management
CMS: Centers for Medicare and Medicaid Services
NLP: natural language processing
PAP: patient assistance program
SDOH: social determinants of health
STROBE: Strengthening the Reporting of Observational Studies in Epidemiology
